# Transcriptome analysis of wheat spikes in response to *Tilletia controversa* Kühn which cause wheat dwarf bunt

**DOI:** 10.1038/s41598-020-78628-0

**Published:** 2020-12-09

**Authors:** Zhaoyu Ren, Jianjian Liu, Ghulam Muhae Ud Din, Han Zhang, Zhenzhen Du, Wanquan Chen, Taiguo Liu, Jianmin Zhang, Sifeng Zhao, Li Gao

**Affiliations:** 1 State Key Laboratory for Biology of Plant Disease and Insect Pests, Institute of Plant Protection, Chinese Academy of Agricultural Sciences, Beijing, 100193 China; 2 School of Agriculture, Yangtze University, Hubei, 434025 China; 3 Key Laboratory at Universities of Xinjiang Uygur Autonomous Region for Oasis Agricultural Pest Management and Plant Protection Resource Utilization, Shihezi University, Xinjiang, 832003 China

**Keywords:** Developmental biology, Microbiology, Plant sciences

## Abstract

Wheat dwarf bunt is caused by *Tilletia controversa* Kühn, which is one of the most destructive diseases of wheat worldwide. To explore the interaction of *T. controversa* and wheat, we analysed the transcriptome profile of spikes of the susceptible wheat cultivar Dongxuan 3, which was subjected to a *T. controversa* infection and a mock infection. The results obtained from a differential expression analysis of *T. controversa*-infected plants compared with mock-infected ones showed that 10,867 out of 21,354 genes were upregulated, while 10,487 genes were downregulated, and these genes were enriched in 205 different pathways. Our findings demonstrated that the genes associated with defence against diseases, such as PR-related genes, WRKY transcription factors and mitogen-activated protein kinase genes, were more highly expressed in response to *T. controversa* infection. Additionally, a number of genes related to physiological attributes were expressed during infection. Three pathways were differentiated based on the characteristics of gene ontology classification. KEGG enrichment analysis showed that twenty genes were expressed differentially during the infection of wheat with *T. controversa*. Notable changes were observed in the transcriptomes of wheat plants after infection. The results of this study may help to elucidate the mechanism governing the interactions between this pathogen and wheat plants and may facilitate the development of new methods to increase the resistance level of wheat against *T. controversa*, including the overexpression of defence-related genes.

## Introduction

Wheat (*Triticum aestivum L*.) is an important staple food crop in most agricultural regions^1,2^. Wheat crops are affected by many fungal diseases, among which dwarf bunt of wheat (DBW) caused by *Tilletia controversa* Kühn is considered to be very dangerous in the majority of wheat-cultivating regions worldwide^3,4^. In the past decade, DBW has devastated several hundreds of acres (1 hectare = 2.471 acres) of wheat in cold areas of the world^5–9^. A typical symptom of DBW infection is the replacement of grains with bunt sori, which contain millions of dark black teliospores. The infected grains have reduced quality and market ability, due to the characteristic odour of trimethylamine^10,11^. Also, the minced flour from DBW-infected grains has a bad taste and fishy smell^12–14^. Because of the quarantine importance of DBW in many countries worldwide regarding wheat production^15,16^_,_ most studies have focused on pathogen detection^17–21^. Assessments of the risk of establishing the disease in China was also reported^22,23^, and these studies mentioned that there were several very high-risk zones in the main winter wheat growing regions, including the northern Xinjiang Uygur Autonomous Region, Tibet, Sichuan, Henan, Shandong, and the Huaihe River Valley. However, few studies have investigated the molecular mechanism governing the interactions of wheat with *T. controversa*. The primary control measure taken against DBW was the use of disease-free grains for sowing and the application of fungicides^16^. Thus, breeding wheat varieties with durable resistance to DBW is one of the most environmentally friendly approaches and may be the most beneficial and effective strategy for disease management, but the development of resistant wheat varieties is laborious, difficult, and time-consuming. Several preliminary genetic studies have been performed, but they have resulted in the discovery of only a small number of genes contributing to control of DBW in field conditions^5^.

Characterizing the response of wheat to *T. controversa* infection is important for determining the mechanisms of wheat resistance to *T. controversa* infection and developing suitable approaches for DBW control. Multilayering occurs between pathogens and plants, and considerable research has been conducted to elucidate the molecular mechanism underlying the interactions between pathogens and plants^24,25^. With advances in plant science, plants have been determined to have many different novel strategies to defend themselves against various levels of invaders, including bacteria, viruses and different classes of fungi^26,27^. These novel strategies of plants contain more complex and linked mechanisms, which increase the correlation of plant pathogen interactions, namely, effector triggered immunity (ETI) and PAMP-triggered immunity (PTI)^28–33^, and some important plant hormones, including salicylic acid, jasmonic acid and ethylene^34–37^. PTI is the initial response of plants under pathogen attack when host receptors diagnose the pathogen-derived pathogen-associated molecular pattern (PAMP), whereas ETI is triggered by the interface between a “resistance” protein and a pathogenic effector^38–40^. Meanwhile, the response to fungal infection of wheat is very complex, and many morpho-physiological processes are involved, but system-level transcriptomic studies may enhance our basic understanding of the response of the host to pathogens. High-throughput gene expression analysis using the most advanced molecular biological technique (RNA-Seq) represents a very powerful tool for transcriptomic characterization of plants during the interaction of pathogens and plants^41,42^. RNA-Seq has been performed successfully in many other crops and pathogens, including tomato and *Xanthomonas perforans* race T3^43^, the chestnut and *Cryphonectria parasitica* strain EP155^44^, potato and *Phytophthora infestants*^45^, banana and *Fusarium oxysporum* f. sp. *cubense*^46^, peach and *Xanthomonas arboricola* pv. *pruni*^47^, soybean and *Fusarium oxysporum*^48^ and mango and *Fusarium mangiferae*^49^. Previous studies demonstrated that several genes were involved in defence mechanisms and resistance-associated signal transduction during plant pathogen interactions^50^. In this study, RNA-Seq was performed to analyse the changes in gene expression and signal transduction in response to *T. controversa* infection. Differentially expressed genes (DEGs) involved in resistance to DBW were investigated after successful infection with *T. controversa*. This approach has led to a greater understanding of the cellular and complex molecular events associated with DBW and provided a basis for further studies on biotechnology and breeding for resistance to DBW disease^51,52^. Many studies have been performed in susceptible cultivars of various crops to examine gene expression over a time course after pathogen infection^53^. DBW is a quarantine significant disease that causes severe losses under optimum conditions in wheat. However, very little is known regarding the molecular and cellular mechanisms underlying the interactions between wheat and *T. controversa*. This study was performed to elucidate the interactions between wheat and *T. controversa* to develop effective strategies for controlling important wheat diseases. To the best of our knowledge, this study is the first to determine the transcriptome changes in wheat after *T. controversa* infection.

## Materials and methods

### Fungal materials and culture

The *T. controversa* isolate was provided by Blair Goates, the United States Department of Agriculture (USDA), Agricultural Research Service (ARS), Aberdeen, Idaho, USA. We purified the isolate using a single isolation method and identified the isolate as race 2, which was highly aggressive. The cultivation methods for the hyphae of *T. controversa* used for inoculation were prepared according a previously described protocol^54^.

### Inoculation of wheat plants with *T. controversa*

Dongxuan 3, a winter wheat cultivar that is highly susceptible to *T. controversa*, was used in this study. Seeds were surface-sterilized with 30% NaClO for 1 min, washed with sterile water 3 times and kept in plates with moist filter paper at 5 °C for one month to vernalize. After vernalization, seedlings were transplanted into pots filled with organic matter and soil at a ratio of 1:2% and were grown in growth chambers (Percival, ARC-36VL-LT, USA). Wheat seedlings were grown in a 14 h light/10 h dark cycle at 5 °C at the tillering stage and at 25 °C at the boot stage. At the early boot stage, when the young tassels of wheat were still wrapped by leaf sheaths, the spikes were injected with 1 ml inoculum suspensions of *T. controversa*. The suspensions contained infectious hyphae at a concentration of 10^6^ cfu/ml and had an OD600 of 0.15. Inoculation was repeated 3 times after a one-day interval. For the mock infection, plants injected with sterilized ddH_2_O were grown under the same conditions. The samples (with spikes measuring 6.0 ± 0.5 cm in length) were collected from both *T. controversa*-infected and mock-infected plants (Supplementary Fig. 1), with three biological replicates being employed for each treatment. Six samples were collected and stored immediately at -80 °C for further use.

### Extraction and purification of RNA

Total RNA was extracted based on the manufacturer’s protocol of the mirVana miRNA Isolation Kit (Ambion, TX, USA). RNA concentration was measured with a Qubit RNA Assay Kit in a Qubit 3.0 Fluorometer (Life Technology, CA, USA). The samples that exhibited an A260/A280 of 1.8 to 2.1 and an A260/A230 > 2.0 were chosen for further analysis. Furthermore, the integrity of each sample was assessed using an Agilent 2100 Bioanalyzer (Agilent Technologies, Santa Clara, CA, USA).

### Library preparation for RNA-Seq and sequencing

Total RNA (1 µg) of each sample of mock- and *T*. *controversa*-infected plants was analysed for library construction. The mRNA was purified by using oligo (dT) magnetic beads, and sequencing libraries were generated with the TruSeq RNA Sample Preparation Kit (Illumina, San Diego, CA, USA) by following all instructions mentioned. The mRNAs were crushed into very small fragments under high temperature in the presence of a fragmentation buffer solution. First-strand complementary DNA was obtained using a solution of random oligonucleotides and SuperScript II reverse transcriptase (Illumina, San Diego, CA, USA); similarly, second-strand complementary DNA was obtained using RNase H and DNA polymerase. A QIAquick PCR kit was employed for purification of cDNA fragments. Then, these cDNA fragments were washed with EB buffer for the addition of end-repair poly (A) and ligated with special sequencing adapters. The final cDNA library was constructed by purification of the cDNA small fragments, which were enriched by PCR products.

### Library examination and sequencing

The constructed cDNA library was validated by using the Qubit RNA Assay Kit in Qubit 3.0 for initial quantification. The insert size was determined using a Bioanalyzer 2100 Agilent system (Agilent, Santa Clara, CA, USA). Furthermore, the insert was amplified using qPCR (7500, ABI, USA). The clustering of every sample was performed on Generation systems (Illumina, USA) following a previously described protocol. The prepared library was loaded onto an Illumina HiSeq X Ten platform with 150-bp paired-end technology.

### Quality control and mapping

The raw data of this experiment were further processed using Trimmomatic (trimmer for Illumina sequence data, Version 0.32)^55^. The reads containing adapter sequences and reads with low quality (those in which more than 50% of bases presented quality of ≤ 10) and poly-N (unrecognized bases) were removed to obtain clean reads. Every downstream analysis was performed based on clear data with significantly high quality. The clean reads were mapped to the reference genome (https://www.ebi.ac.uk/ena/data/view/GCA_900519105.1) using hisat2^56^ with the parameters set by the system.

### Gene-level quantification and identification of differentially expressed genes (DEGs)

The FPKM value of every gene was analysed and calculated by using cufflinks (version 2.2.1)^57,58^, and every read count of all genes was obtained by HTSeq-count^59^. Additionally, the DEGs of this study were recognized by using the DESeq^60^ technique. Furthermore, the FDR ˂ 0.05, and at least a two-fold change (> 1 or < − 1 in log_2_ ratio value) was set as the threshold for DEGs. Hierarchical cluster analysis (HCA) of all DEGs was performed to explore gene expression patterns.

### Kyoto Encyclopedia of Genes and Genomes (KEGG) and gene ontology (GO) enrichment analysis

KEGG (https://www.kegg.jp/kegg/kegg1.html) pathway analysis was performed by using GPSeq, and GO enrichment analysis was performed, with FDR < 0.05 representing the significantly expressed genes^61,62^.

### Validation of RNA-Seq results by quantitative real-time PCR analysis

Eight transcripts with various expression levels demonstrated by RNA sequencing were randomly selected for proof by qRT-PCR. Total RNA was extracted from three *T. controversa*-infected spikes and three mock-infected spikes. First-strand cDNA was synthesized by using 2000 ng µl^−1^ purified total RNA, RT/RI enzyme and oligo (dT)18 primer (TransGen Biotech, Beijing, China) following the instructions of the manufacturer. The primers employed in this experiment are listed in Table S1. Actin was used as an internal control in this experiment. RT-qPCR was performed using Top Green qPCR SuperMix (TransGen, China) in a volume of 20 µL by following the instructions of the manufacturer and applied to the QuantStudio 5 instrument, which was part of a real-time PCR system (Applied Biosystems, Beijing, China). Three technical replicates were employed for every gene. All genes were run on a 96-well QuantStudio 5 (Applied Biosystems, Beijing, China) with the following conditions: pre-denaturation at 95 °C for 10 min and 40 cycles (95 °C for 15 s, 58 °C for 30 s and 72 °C for 30 s). The 2^−ΔΔCt^ method^63^ was employed to calculate the expression level of every gene.

## Results

### Confirmation of *T. controversa* infection in wheat plants

After inoculation with *T. controversa*, symptoms appeared in the spike with the formation of black teliospores. Specifically, the heads of infected plants were thicker and wider and squarrose. The florets were filled with bunt balls (sori). These bunt balls replaced the grains and produced black teliospores with a rotten fish-like odour. To identify the genes with changed expression following infection by *T. controversa* and to detect any change in gene expression levels after infection, RNA extraction was performed to investigate the changes in wheat at the transcriptomic level.

### Transcriptomic analysis of RNA-Seq data

**Figure 1:**
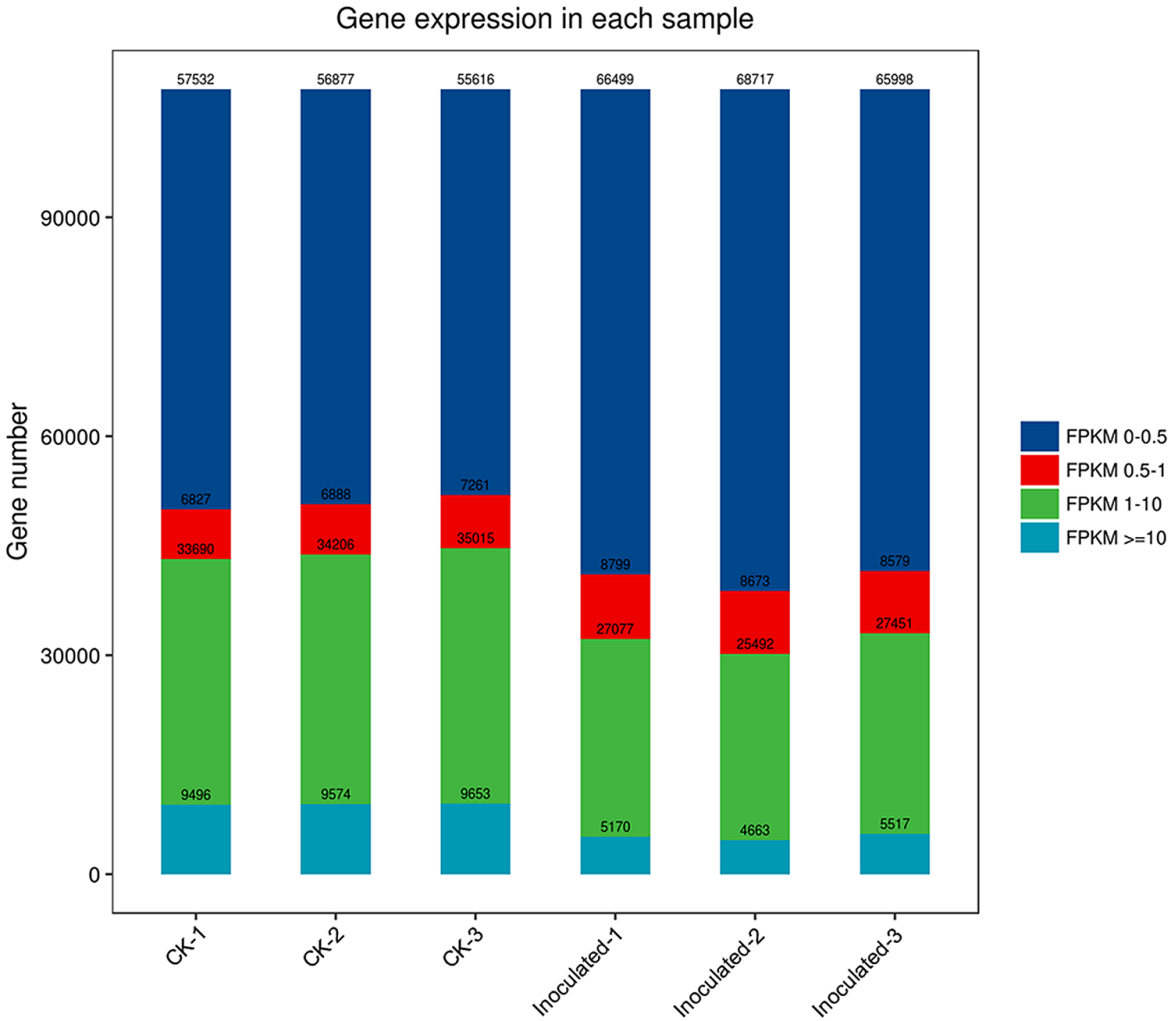
Summary of differentially expressed genes (DEGs). Numbers of DEGs between CK and *T. controversa* infection. CK indicates mock plants, and Inoculated indicates plants infected by *T. controversa.*

**Figure 2:**
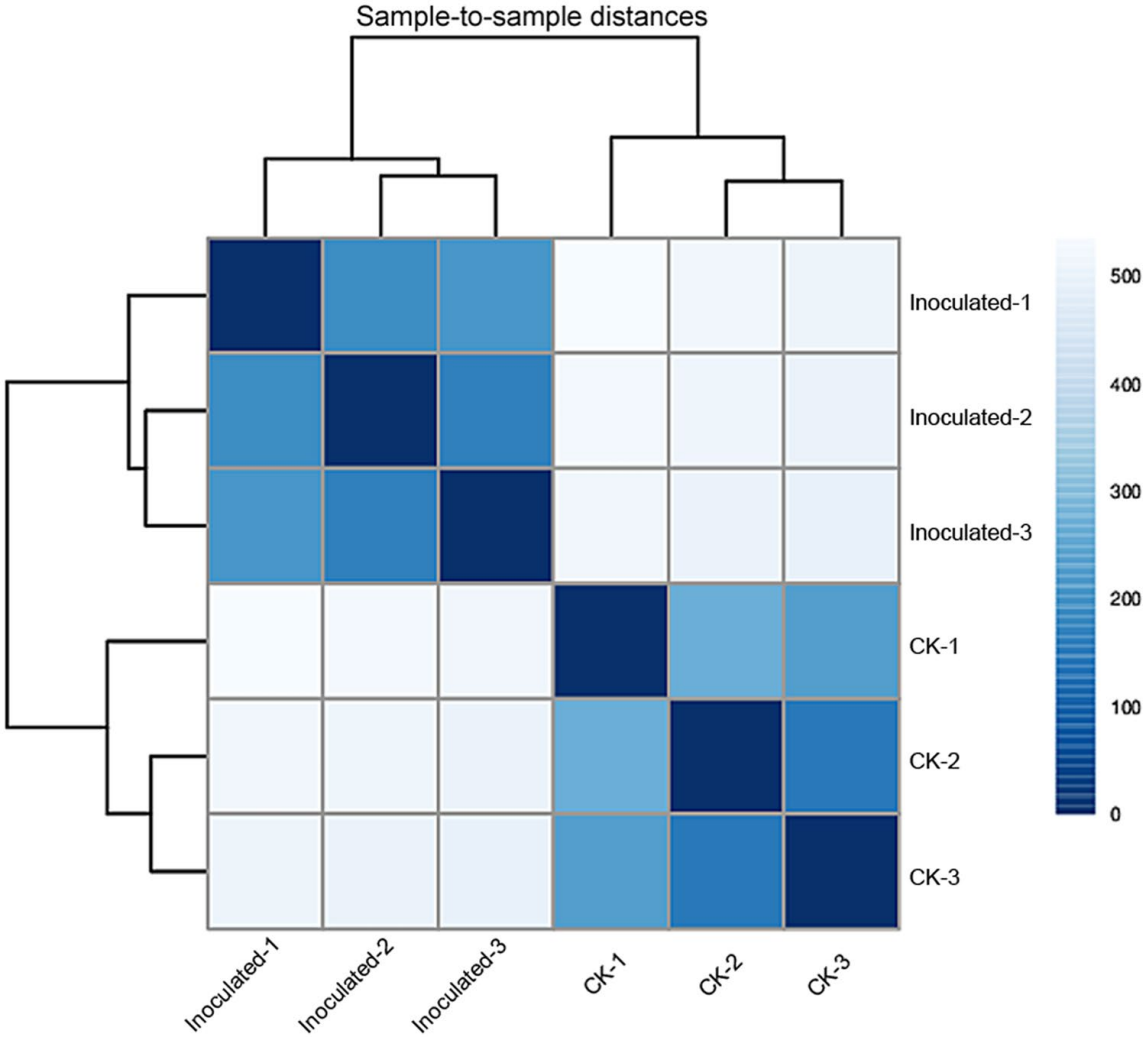
Sample-to-sample clustering analysis for checking batch effects and their similarity. CK indicates mock plants, and Inoculated indicates plants infected by *T. controversa.*

**Figure 3:**
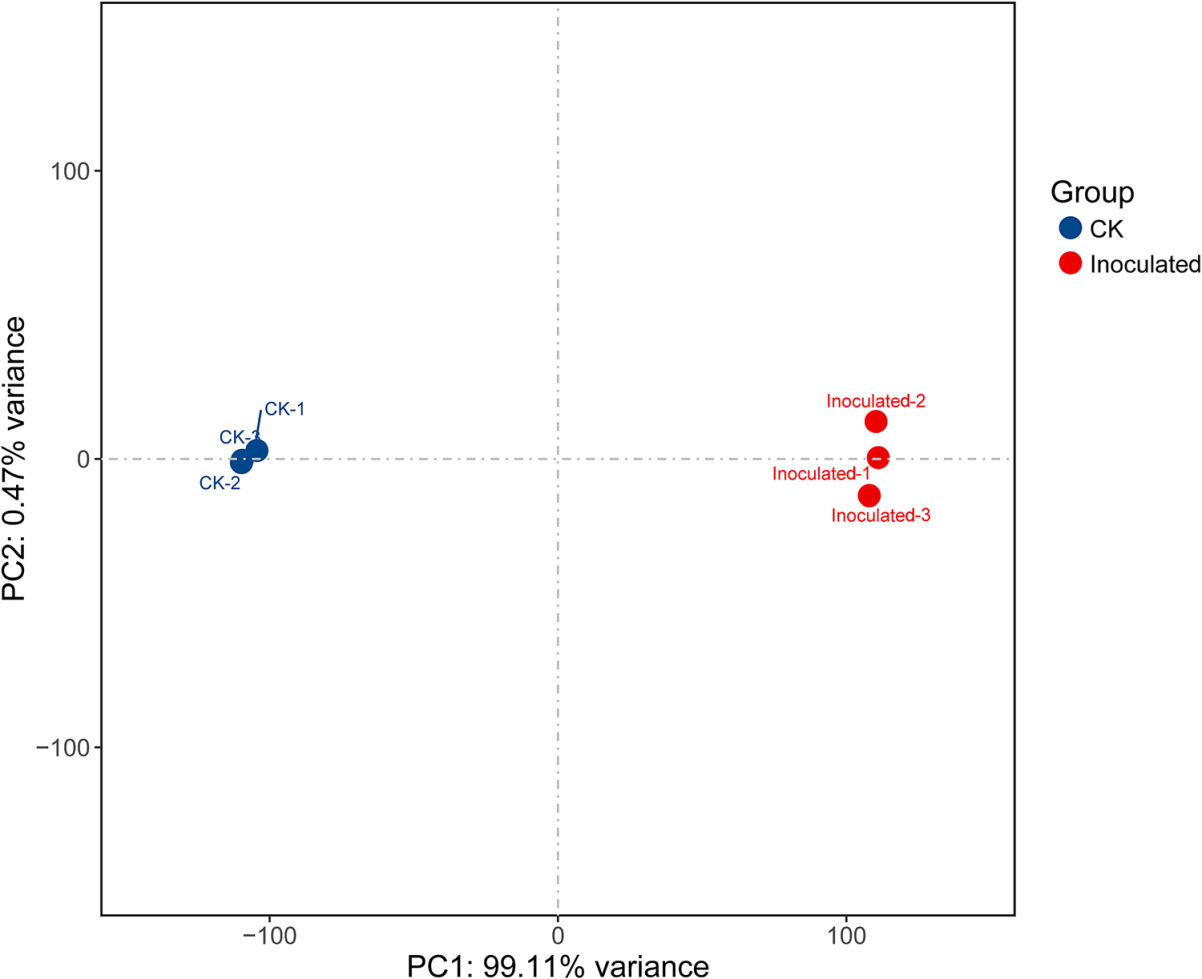
Principal component analysis (PCA) for gene expression patterns. The first and second PCAs explained 99.11% and 0.47% of the variance, respectively. CK indicates mock plants, and Inoculated indicates plants infected by *T. controversa.*

Based on RNA-Seq, we identified alterations in wheat genes when the spike was infected by *T. controversa*. Six cDNA libraries (three *T. controversa*-infected and three mock-infected) were sequenced. Raw reads were trimmed by removing empty reads, adaptor sequences and low-quality sequences. Approximately 55.95, 50.30 and 52.45 million raw reads were obtained from the mock-infected plants CK-1, CK-2 and CK-3, respectively, and raw reads in *T. controversa*-infected plants were 58.54, 58.50 and 53.72 million in *T. controversa* (Inoculated-1), *T. controversa* (Inoculated-2) and *T. controversa* (Inoculated-3), respectively. Similarly, 54.55, 49.23 and 51.11 million clean reads (high-quality reads) were obtained from mock-infected plants, and 55.72, 55.55 and 51.04 million reads were obtained from Inoculated-1, Inoculated-2 and Inoculated-3, respectively. Multiple and unique maps of the abovementioned transcripts ranged from 7.05 to 11.80% and 79.56 to 87.05%, respectively. Additionally, the Q30 and GC of these transcripts ranged from 94.15 to 96.02% and 50.84 to 55.66%, respectively (Table 1). Next, the differentially expressed genes were recognized by comparing the FPKM value of every gene between CK and *T. controversa* (Inoculated) samples. For CK-1 DEGs, 9496 (FPKM ˃ = 10), 33,690 (FPKM 1–10), 6827 (FPKM 0.5–1), and 57,532 (FPKM 0.–0.5) genes were differentially expressed. An approximately similar response was observed in CK-2 and CK-3 samples. For Inoculated-1 DEGs, 5150 (FPKM ˃ = 10), 27,077 (FPKM 1–10), 8799 (FPKM 0.5–1), and 66,499 (FPKM 0.-0.5) genes were differentially expressed. The differential expression of genes in CK and *T. controversa* indicates the genetic difference between mock- and *T. controversa*-infected plants (Fig. 1). Additionally, three biological replicates of every sample were clustered together. Sample-to-sample clustering analysis demonstrated that the gene expression level between replicates was reproducible and that batch effects were controlled (Fig. 2). Furthermore, principal component analysis (PCA) was performed for both mock- and *T. controversa*-infected samples. The mock was located around the junction of the second and third quadrants, and the *T. controversa* infection was located around the junction of the first and fourth quadrants, indicating that there is good reproducibility among the biological replicates of the same treatments but differences between the treatments (Fig. 3).

**Table 1:** Transcriptome analysis of RNA-Seq data.

Type	CK-1	CK-2	CK-3	Inoculated-1	Inoculated-2	Inoculated-3
Raw reads	55.95 M	50.30 M	52.45 M	58.54 M	58.50 M	53.72 M
Clean reads	54.55 M	49.23 M	51.11 M	55.72 M	55.55 M	51.04 M
Multiple mapped	3,846,328 (7.05%)	3,561,802 (7.23%)	3,703,289 (7.25%)	6,329,075 (11.36%)	6,553,508 (11.80%)	5,539,728 (10.85%)
Unique mapped	47,120,475 (86.38%)	42,857,444 (87.05%)	44,404,427 (86.87%)	44,779,099 (80.37%)	44,195,418 (79.56%)	41,369,664 (81.06%)
Q30	95.81%	96.02%	95.73%	94.35%	94.25%	94.15%
GC	50.69%	50.84%	51.08%	54.95%	53.77%	55.66%

CK stands for mock and Inoculated stands for *T. controversa* inoculated plants. Numbers represent replication of cDNA libraries generated for respective samples.

### Identification of differentially expressed genes (DEGs)

**Figure 4:**
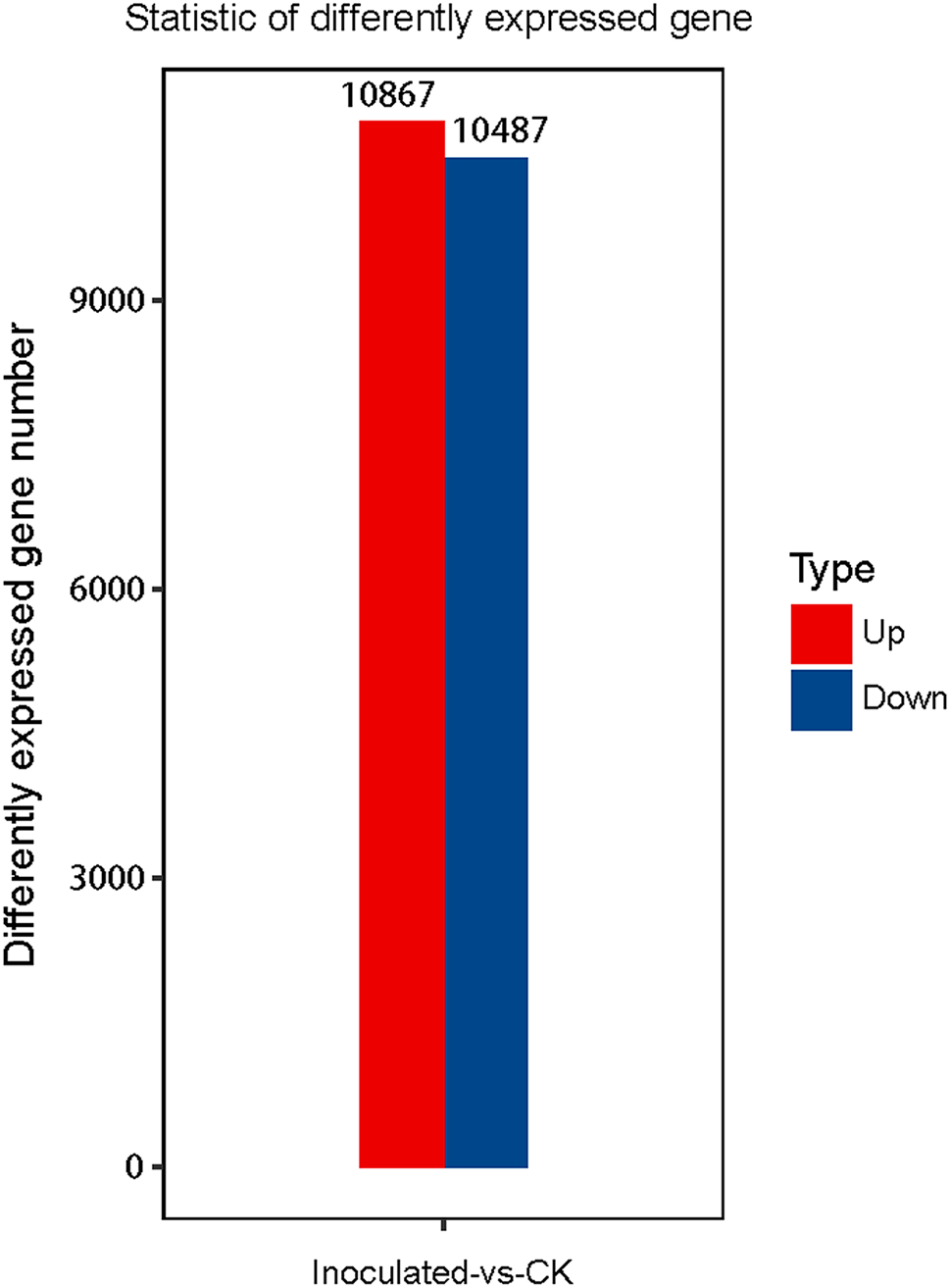
Significant differentially expressed genes (DEGs) in *T. controversa*-infected vs. mock libraries. Up- or downregulated DEGs in response to *T. controversa* infection. CK indicates mock plants, and Inoculated indicates plants infected by *T. controversa.*

**Figure 5:**
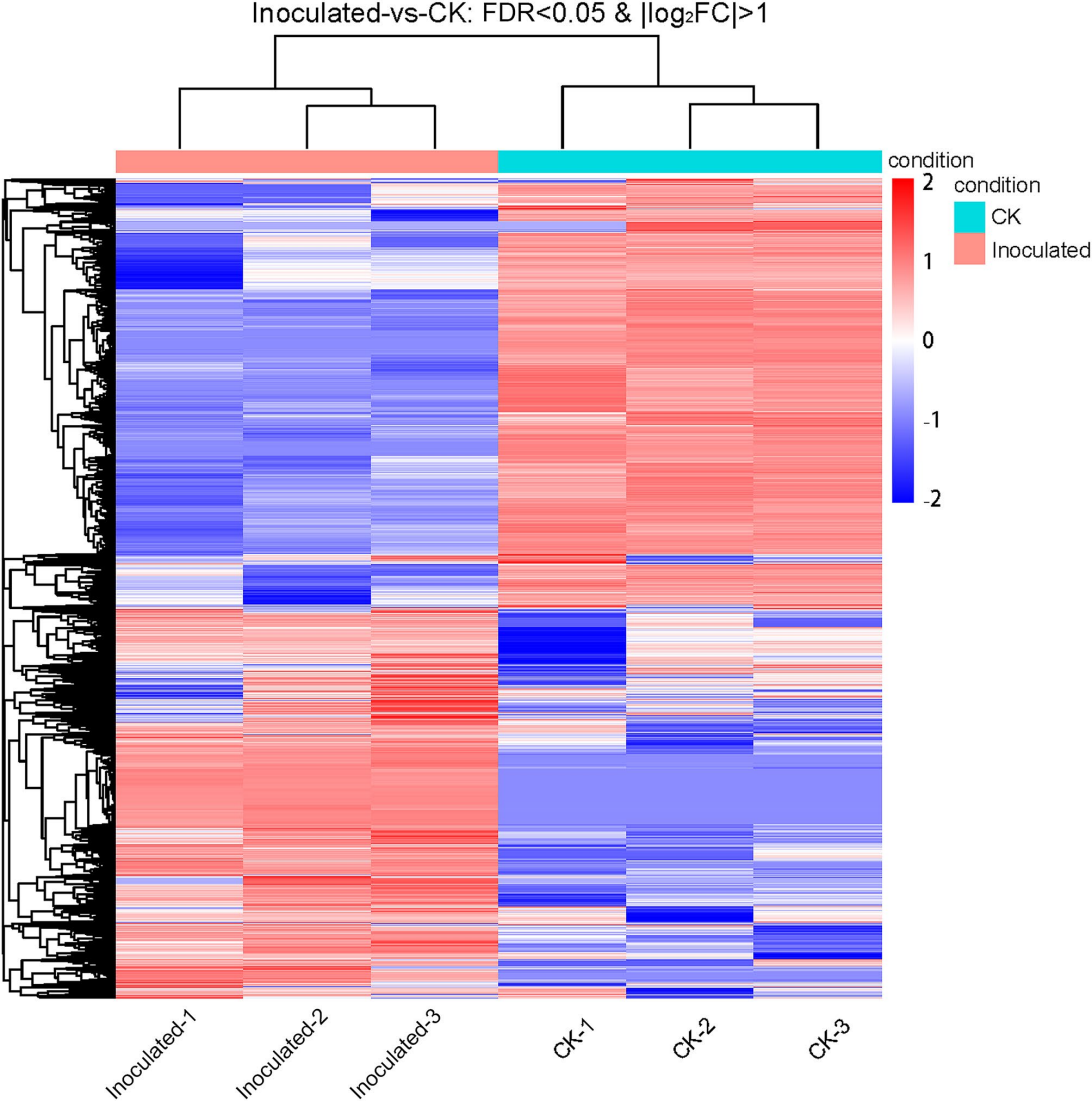
Hierarchical clustering heatmap of DEGs according to changes in expression in response to *T. controversa* infection. Each column shows a library, and each row shows DEG expression. The colours blue, white and red indicate low, medium, and high expression patterns of genes, respectively. CK means the mock plants, inoculated mean the plants were infected by *T. controversa.*

The differentially expressed genes were recognized in *T. controversa-*infected and mock-infected libraries. In this comparison, 10,867 (up-regulated) and 10,487 (down-regulated) genes were expressed (Fig. 4; Table S2). To elucidate the transcriptional changes occurring after *T. controversa* infection, we demonstrated the expression pattern by using hierarchical clustering analysis. On behalf of the analysis, the expression levels of *T. controversa*-infected and mock-infected plants were different from each other but were similar in the replication of *T. controversa*-infected and mock-infected plants. There were more up-regulated genes than down-regulated genes in *T. controversa*-infected plants (Fig. 5).

### Gene ontology (GO) enrichment analysis of DEGs

**Figure 6:**
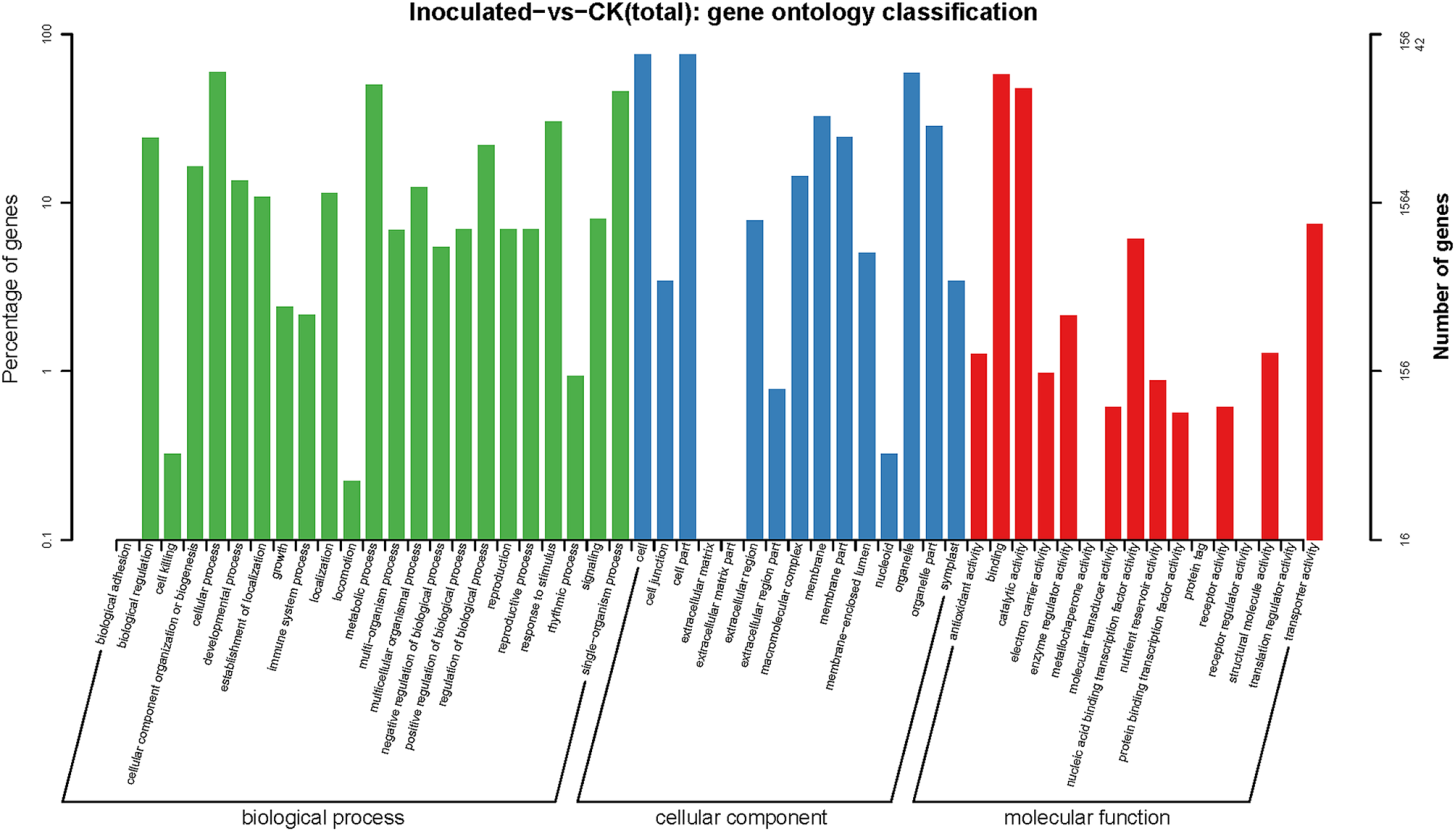
Gene ontology (GO) enrichment analysis of significant DEGs of *T. controversa*-infected and mock libraries. Annotations are grouped by biological process, cellular component, and molecular function. CK indicates mock plants, and Inoculated indicates plants infected by *T. controversa.*

GO enrichment analysis of DEGs can demonstrate the function of genes. GO was categorized into three main domains based on their functions: biological process, cellular component and molecular function. Our results showed that in the biological process category, during the comparison of *T. controversa*-infected and mock-infected plants, GO was mainly associated with cellular process, metabolic process, multicellular organismal process, regulation of biological process, reproduction, reproduction process, response to stimulus, and single-organism process. Meanwhile, in the cellular component category, DEGs were primarily associated with cell, cell part, extracellular region, macromolecular complex, membrane, membrane part, organelle, and organelle part. In the molecular function category, DEGs primarily mapped with binding, catalytic activity, enzyme regulator activity, nucleic acid binding transcription factor activity, structural membrane activity and transporter activity (Fig. 6). In contrast, biological adhesion, cell killing and locomotion were observed to be uniquely enriched in biological process, while extracellular matrix, extracellular matrix part and nucleoid were uniquely enriched in cellular component and metallochaperone activity, protein tag, receptor regulator activity and translation regulator activity were determined to be uniquely enriched in molecular function (Fig. 6).

### KEGG enrichment analysis of DEGs

**Figure 7:**
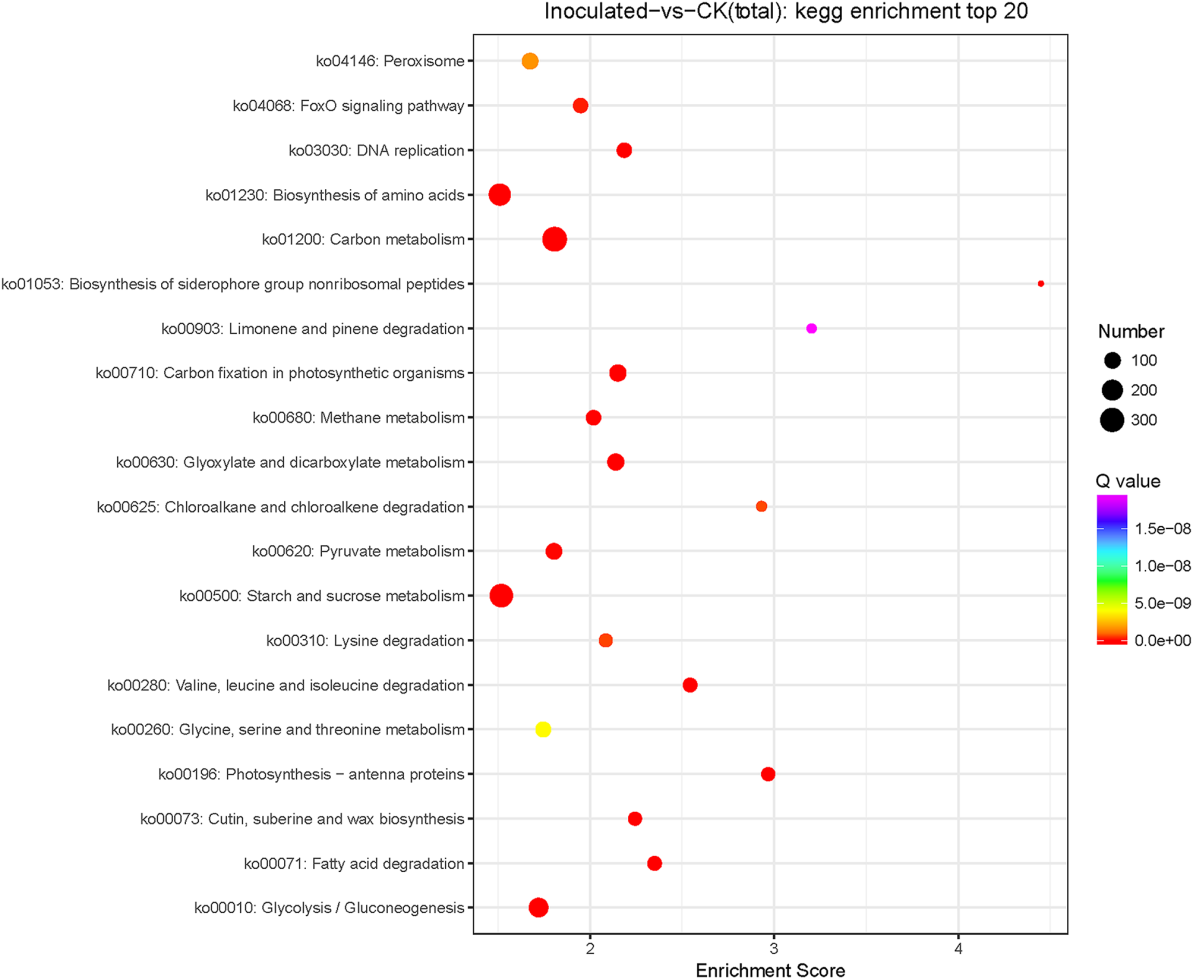
KEGG enrichment analysis scatter plot representing pathways of DEGs in response to *T. controversa* infection. The colours blue, white and red indicate low, medium, and high expression patterns of genes, respectively. CK indicates mock plants, and Inoculated indicates plants infected by *T. controversa.*

KEGG analyses were performed to better understand the molecular associations among the DEGs. For DEGs between *T. controversa*-infected and mock-infected plants, 205 different pathways were identified (Table S3). However, the top 20 KEGG enrichment pathways of peroxisomes, FoxO signalling pathway, DNA replication, biosynthesis of amino acids, carbon metabolism, carbon fixation in photosynthetic organisms, methane metabolism, glyoxylate and dicarboxylate metabolism, chloroalkane and chloroalkene degradation, pyruvate metabolism, starch and sucrose metabolism, lysine degradation, valine, leucine and isoleucine degradation, glycine-serine-and threonine metabolism, photosynthesis-antenna proteins, cutin, suberine and wax biosynthesis, fatty acid degradation and glycolysis/gluconeogenesis were primarily activated. The pathway of biosynthesis of siderophore group nonribosomal peptides was activated slightly during the interaction (Fig. 7).

### Differential expression of pathogenesis-related genes after *T. controversa* infection

Previous studies have shown that PR genes encoding 1,3-glucanases, chitinases and thaumatin-like proteins enhanced the resistance of wheat against various fungal pathogens^64^. By comparing the transcriptome results obtained in *T. controversa*-infected plants with those of mock-infected cells, it was found that the transcriptional level of PR genes changed. The results showed that 7 pathogenesis-related genes, 23 thaumatin-like genes, 28 chitinase genes, 121 peroxidase genes, and 36 glucanase genes changed during *T. controversa* infection. Most of these genes were up-regulated (Table S4).

### Differential expression of WRKY transcription factors after *T. controversa* infection

Following *T. controversa* infection, we identified significant DEGs of WRKY transcription factors in *T. controversa*-infected libraries compared with mock-infected ones. Most WRKY transcription factors showed up-regulation after *T. controversa* infection. The results showed that 44 out of 57 and 13 out of 57 WRKY transcription factors were up-regulated and down-regulated, respectively (Table S5).

### Differential expression of protein kinase genes after *T. controversa* infection

Following *T. controversa* infection, we identified significant DEGs of protein kinase genes in *T. controversa*-infected libraries compared to mock-infected libraries. The results showed that 29 calcium-dependent protein kinases, 35 CBL-interacting protein kinases, 7 cold-responsive protein kinases, 11 cyclin kinases, 58 cysteine-rich receptor-like protein kinases, 48 G-type lectin S-receptor-like serine/threonine-protein kinases, 26 leucine-rich repeat receptor-like protein kinases, 21 LRR receptor-like serine/threonine-protein kinases, 50 mitogen-activated protein kinases, 7 probable inactive leucine-rich repeat receptors, 21 probable leucine-rich repeat receptor-like protein kinases, 55 probable LRR receptor-like serine/threonine-protein kinases, 22 probable receptor-like protein kinases**,** 66 probable serine/threonine-protein kinases, 10 proline-rich receptor-like protein kinases, 8 putative cysteine-rich receptor-like protein kinases, 9 receptor protein kinases, 143 serine/threonine-protein kinases, 10 shaggy-related protein kinases, 15 SNF1-related protein kinases and 110 changed after infection (Table S6).

### Quantitative real-time PCR

To verify the changes in expression level exhibited by the identified DEGs in response to *T. controversa* infection, the expression levels of eight genes examined by quantitative real-time PCR (qRT-PCR). The expression pattern of validated genes was similar to the results obtained from RNA-Seq (Table 2). The qRT-PCR results showed that seven genes were up-regulated and Lipase was determined to be down-regulated by both RNA-Seq and qRT-PCR analyses. Hence, the qRT-PCR results confirmed the RNA-Seq data.

**Table 2:** Validation of RNA-Seq data by quantitative real-time PCR (qRT-PCR). Expression level of selected DEGs between mock- and *T. controversa*-infected libraries.

Gene ID	Genes annotation		FDR	FPKM	qRT-PCR	Validated
TraesCS3A02G525700	Pathogenesis-related protein-1		2.67E-03	Inf (very low)	1.26 ± 0.21 up	Yes
TraesCS7D02G351300	Chitinase 1		4.35E-09	5.93 up	4.78 ± 0.32 up	Yes
TraesCS1D02G249600	Chitinase 2		3.48E-04	5.26 up	3.21 ± 0.11 up	Yes
TraesCS2B02G369000	Chitinase 4		2.62E-07	3.02 up	2.34 ± 0.10 up	Yes
TraesCS3D02G227400	WRKY22		4.09E-02	3.16 up	2.33 ± 0.60 up	Yes
TraesCS1A02G348600	WRKY24		4.29E-03	Inf (very low)	0.96 ± 0.07 up	Yes
TraesCS1A02G094700	Lipase		3.10E-02	-3.08 down	−2.02 ± 0.02 down	Yes
TraesCS1A02G249600	Endo-1,4-beta-glucanase		5.37E-03	1.09 up	0.94 ± 0.14 up	Yes

## Discussion

In the earliest studies of *T. controversa* and wheat, microscopic studies were performed to observe the structural changes in wheat after infection. In this research, we examined the plant defence responses in wheat following infection by *T. controversa*. We investigated interactions between susceptible wheat cultivars (Dongxuan 3) and *T. controversa*. We hypothesized that in these interactions, wheat transcriptomic changes are associated with the plant response to infection; thus, diseases appeared. Therefore, in this experiment, we employed RNA-Seq to perform a transcriptomic study of wheat following *T. controversa* infection and analysed the changes in the expression levels of genes in mock- and *T. controversa*-infected wheat plants. Our results demonstrated significantly differentially expressed genes between mock- and *T. controversa*-infected libraries.

Pathogenesis-related (PR) genes play a key role in the defence mechanisms of plants against biotic factors^64–67^. Overexpression of the PR genes encrypting pathogenesis-related proteins, thaumatin-like proteins, chitinases, peroxidases and glucanases increases resistance to various pathogens in different crops^11,68,69^. We compared the transcription level between pathogen-infected and mock-infected plants at the flowering stage. The transcription levels of 215 PR genes were changed by *T. controversa* infection (Table S4), including seven pathogenesis-related proteins, twenty-three thaumatin-like proteins, twenty-eight chitinase proteins, one hundred twenty-one peroxidase proteins and thirty-six glucanase proteins. Most of the one hundred thirty-five genes were up-regulated, and eighty were down-regulated. Together, these defence-related proteins might play a role in disease suppression against *T. controversa*.

WRKY transcription factors represent the largest protein family in plants, can activate different defence mechanisms and play pivotal roles in regulating defence genes^35,70–74^. DEG analysis of *T. controversa*-infected libraries versus mock-infected libraries showed that fifty-seven WRKY genes were expressed. Forty-four WRKY transcription factors were up-regulated, while thirteen were down-regulated. Some of the up-regulated WRKY transcription factors, including WRKY transcription factors 23 and 27, play roles in the resistance to biotic diseases (Table S5)^75–78^.

Pattern recognition receptors (PRRs) have been developed to recognize MAMPs/PAMPs (microbe/pathogen-associated molecular patterns), which are conserved small molecules present across a broad classification of microbes. These plant receptors belong to the receptor-like kinase (RLK) family^79–81^. In our study, 761 different protein kinases were expressed in wheat ears after *T. controversa* infection (Table S6). Most kinase proteins were down-regulated after *T. controversa* infection. Interestingly, cysteine-rich receptor-like protein kinase, leucine-rich repeat receptor protein kinase, probable inactive leucine-rich repeat receptor, probable LRR receptor-like serine/threonine-protein kinase, probable receptor-like protein kinase and proline-rich receptor-like protein kinase were almost completely down-regulated after infection. These consequences indicate that *T. controversa* releases and carries effectors into wheat ear cells during attack to overcome the immune signalling pattern of plants, thereby leading to DBW. Our results concerning kinase proteins were similar to those obtained by Hosseini^82^ in their studies of another pathogen. Mitogen-activated protein kinase (MAPK) genes have been investigated in the plant response to fungal pathogens^83^. In our study, we found that fifty MAPK genes were expressed after *T. controversa* infection (Table S6), which suggests that MAPK genes play a role in wheat resistance to *T. controversa* infection.

According to GO enrichment analysis of differentially expressed genes in mock-infected and *T. controversa*-infected libraries, GO was categorized into three main domains based on their functions: biological process, cellular component, and molecular function. (Fig. 6). The results showed that cellular process and metabolic process had the highest number of DEGs during plant pathogen interactions. The GO results support the hypothesis that plant pathogens cause changes in the primary (plant growth and development) and secondary (induction of defence programme) metabolisms of the plants. Once plants are infected by pathogens, plant metabolism tends to expend more energy on plant defence activation compared with growth, development, cellular maintenance and reproduction^84–87^. This phenomenon suggests that the DEG metabolic process is related to the pathogenic mechanism of *T. controversa*, as well as plant-pathogen interactions.

KEGG pathway analysis demonstrated that most DEGs were characterized by carbon metabolism, starch and sucrose metabolism, biosynthesis of amino acids and glycolysis/gluconeogenesis pathways (Fig. 7; Table S3). Biosynthesis of the siderophore group nonribosomal peptide pathway included down-regulated genes that encode ferric iron acquisition in many microorganisms. Iron plays a role in microbial proliferation and growth. Thus, iron are supposed to play a key role in disease development^88,89^. Microbial ferric iron reductase is a key enzyme that degrades ferric iron in microbes^90^. Our results are in keeping with the down-regulation of microbial ferric iron reductase due to fungal infection^91,92^. The results obtained from KEGG analysis of DEGs showed that significant DEGs were annotated to 205 different pathways, which suggests that *T. controversa* infection affects various biological functions of wheat (Table S3). Dwarfing is an important symptom of DBW; changes in gene expression related to morpho-physiological characteristics, especially plant height, are notable in wheat infected by *T. controversa* and might be involved in dwarfing symptoms. Our results showed that the expression of cytochrome P450 changed due to *T. controversa* infection (Table S3). Cytochrome P450 has various biosynthetic activities and plays a positive role in plant growth and development by producing gibberellins and brassinosteroids^93,94^.

Overall, our findings provide a genome-wide gene expression profile for wheat plants infected with *T. controversa* and may help to elucidate the regulatory mechanisms governing the response of wheat to this pathogen.

## Supplementary information

Supplementary Information.

Supplementary Table S1.

Supplementary Table S2.

Supplementary Table S3.

Supplementary Table S4.

Supplementary Table S5.

Supplementary Table S6.
