# Supplementary material for: Transcriptome analysis of wheat spikes in response to Tilletia controversa Kühn which cause wheat dwarf bunt
Source: Sci Rep. 2020 Dec 9;10:21567. doi: 10.1038/s41598-020-78628-0 (PMC7725808; doi:10.1038/s41598-020-78628-0)

Title page for Supplementary Information

**Transcriptome analysis of wheat spikes in response to *Tilletia controversa* Kühn which cause wheat dwarf bunt**

**Zhaoyu Ren^1+^, Jianjian Liu^1,2+^, Ghulam Muhae Ud Din^1+^, Han Zhang^1,3^, Zhenzhen Du^1^, Wanquan Chen^1^, Taiguo Liu^1^, Jianmin Zhang^2^, Sifeng Zhao^3^ and Li Gao^1*^**

*^1^State Key Laboratory for Biology of Plant Disease and Insect Pests, Institute of Plant Protection, Chinese Academy of Agricultural Sciences, Beijing 100193, China.*

*^2^School of Agriculture, Yangtze University, Jingzhou, China.*

*^3^Key Laboratory at Universities of Xinjiang Uygur Autonomous Region for Oasis Agricultural Pest Management and Plant Protection Resource Utilization, Shihezi University, 832003, China*

**^+^**Zhaoyu Ren, Jianjian Liu, Ghulam Muhae Ud Din contributed equally to this manuscript.

**Corresposnding author: Li Gao (xiaogaosx@hotmail.com)**

Table S1. The primers were listed for validation of genes in this experiment

Table S2. Differentially expressed genes in *T. controversa* infected and mock plants.

Table S3. KEGG pathway analysis in *T. controversa* infected and mock plants

Table S4. Differential expression of pathogenesis related genes in plants after *T. controversa* infection

Table S5 Differential expression of WRKY transcription factors in plants after *T. controversa* infection

Table S6 Differential expression of protein kinase in plants after *T. controversa* infection

Figure S1: The picture which showed the confirmation of *T. controversa* infection and the symptoms for the infected spike of wheat. (a) normal wheat spike (b) spike infected by *T. controversa*


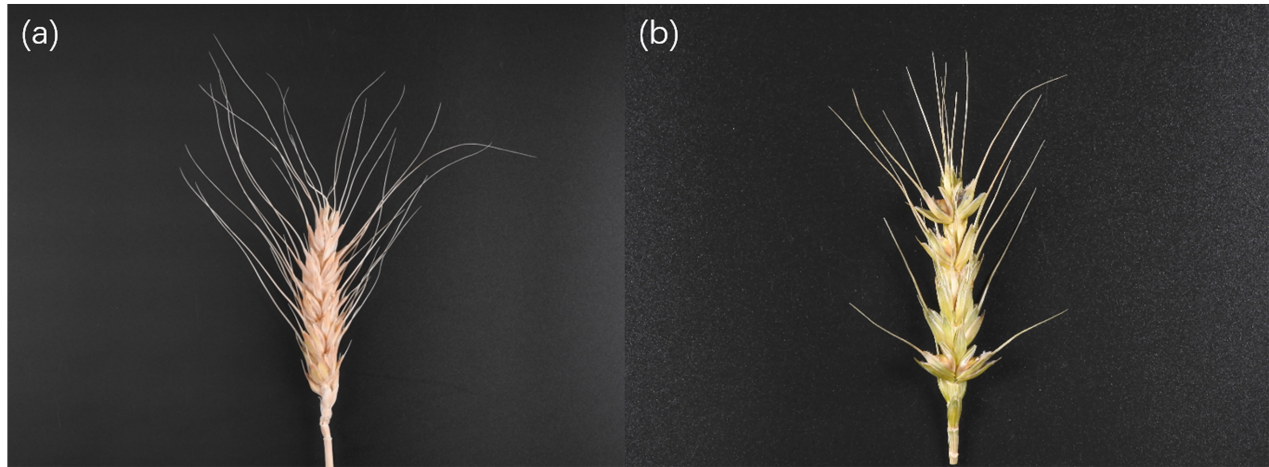

Supplement: Supplementary file 1 — Supplementary Information. [file 41598_2020_78628_MOESM1_ESM.docx]
