# Supplementary material for: Transcriptome analysis of wheat spikes in response to Tilletia controversa Kühn which cause wheat dwarf bunt
Source: Sci Rep. 2020 Dec 9;10:21567. doi: 10.1038/s41598-020-78628-0 (PMC7725808; doi:10.1038/s41598-020-78628-0)
Supplement: Supplementary file 2 — Supplementary Table S1. [file 41598_2020_78628_MOESM2_ESM.docx]

Table S1. The primers were listed for validation of genes in this experiment

| Genes anotation | Primer | | Sequence (5'to3') |
| --- | --- | --- | --- |
| Pathogenesis-related protein-1 | | F | GCCAGCTACTACTCTCTCCG |
| Pathogenesis-related protein-1 | | R | AGGTATCCCATGCACGACTC |
| Chitinase 1 | | F | CTACACGTACGACGCCTTCA |
| Chitinase 1 | | R | GACGTGGCCTTGCTTATCTC |
| Chitinase 2 | | F | CACCCGGCAAGCAGTACTAT |
| Chitinase 2 | | R | ACCATATCGCCGTCTTGAAC |
| Chitinase 4 | | F | TTCTGGTTCTGGATGACCAAC |
| Chitinase 4 | | R | ACTGCTTGCAGTACTCCGTGT |
| WRKY22 | | F | CAAATGGCCGACGATTGGGATCTC |
| WRKY22 | | R | CTAGTCCCCCGCGAATCATA |
| WRKY24 | | F | TTGATGAAACCCTAATGATGATGC |
| WRKY24 | | R | AGATGTTGGGTAGCGGGTTTGACT |
| Lipase | | F | ACTGGGTATTCGTCTGTCAGC |
| Lipase | | R | CACAAAATATCGACCCACCAC |
| Endo-1,4-beta-glucanase | | F | CCTTGCCTCTTTGTATGCTGA |
| Endo-1,4-beta-glucanase | | R | TCATCTTTTGTGGGTTCTTGC |
| Actin | | F | CACTGGAATGGTCAAGGCTG |
| Actin | | R | CTCCATGTCATCCCAGTTG |
